# ITG: A New Global GNSS Tropospheric Correction Model

**DOI:** 10.1038/srep10273

**Published:** 2015-07-21

**Authors:** Yibin Yao, Chaoqian Xu, Junbo Shi, Na Cao, Bao Zhang, Junjian Yang

**Affiliations:** 1 School of Geodesy and Geomatics, Wuhan University, 129 Luoyu Road, Wuhan, 430079, China; 2 Key Laboratory of Geospace Environment and Geodesy, Ministry of Education, Wuhan University, 129 Luoyu Road, Wuhan, 430079, China

## Abstract

Tropospheric correction models are receiving increasing attentions, as they play a crucial role in Global Navigation Satellite System (GNSS). Most commonly used models to date include the GPT2 series and the TropGrid2. In this study, we analyzed the advantages and disadvantages of existing models and developed a new model called the Improved Tropospheric Grid (ITG). ITG considers annual, semi-annual and diurnal variations, and includes multiple tropospheric parameters. The amplitude and initial phase of diurnal variation are estimated as a periodic function. ITG provides temperature, pressure, the weighted mean temperature (Tm) and Zenith Wet Delay (ZWD). We conducted a performance comparison among the proposed ITG model and previous ones, in terms of meteorological measurements from 698 observation stations, Zenith Total Delay (ZTD) products from 280 International GNSS Service (IGS) station and Tm from Global Geodetic Observing System (GGOS) products. Results indicate that ITG offers the best performance on the whole.

Tropospheric delay is a key radio wave propagation effect that contributes to the error budget of GNSS^4^,^5^, containing hydrostatic and wet terms^6^. It can be mapped to zenith tropospheric delay using a mapping function^7^,^8^,^9^,^10^,^11^. Under conditions of static equilibrium, zenith hydrostatic delay (ZHD) can be derived with millimeter-level accuracy from meteorological parameters^12^ and position at an observation site using model^13^. ZWD is used as an estimated parameter for higher accuracy^14^,^15^, and can be transformed into precipitable water vapor (PWV) with Tm^16^. Tm can be derived from measured meteorological parameters. Without measurements, the reference atmosphere is used to calculate Tm. However, this introduces considerable error. Empirical models address this issue.

An early version is a series of UNB models developed by Collins and Langley^17^,^18^ and adopted for Wide Area Augmentation System. Based on the United States Standard Atmospheres, various atmospheric parameters with a latitude interval of 15° can be used to estimate the desired meteorological parameters. The average error of tropospheric zenith delay is about 2 cm for UNB3 in North America, nearly identical to Saastamoinen on a global scale. European Geostationary Navigation Overlay Service^19^ (EGNOS) is used to simplify UNB3 with similar accuracy and it has been widely used in satellite navigation augmentation systems in Europe and Japan^20^,^21^. Based on the output of Numerical Weather Model (NWM) from National Centers for Environmental Prediction (NCEP), Krueger *et al.*^22^ established TropGrid with a horizontal resolution of 1° × 1°. TropGrid improves accuracy by a global average of 25% compared with EGNOS.

Based on the output of NWM from European Centre for Medium-Range Weather Forecasts (ECMWF) ERA-40, Boehm *et al.*^23^ established an empirical model called Global Pressure and Temperature (GPT) for pressure and temperature^24^. GPT expresses results in terms of spherical harmonics with nine degrees and nine orders. The model has been widely applied^25^,^26^. Lagler *et al.*^1^ proposed a new model, GPT2, to improve some weaknesses of GPT. GPT2 is based on a more precise ECMWF ERA-Interim, and adds semi-annual variation to estimate the initial phase of each cycle. Results are expressed with a horizontal resolution of 5° × 5° rather than spherical harmonics, resulting in higher horizontal resolution. TropGrid2 is an enhanced version of TropGrid, developed by Schüler^3^, providing temperature, pressure, Tm, ZWD and other key tropospheric parameters. Boehm *et al.*^2^ established GPT2w, which adds the water vapor lapse rate and Tm and improves horizontal resolution to 1° × 1° compared to GPT2.

The development of tropospheric correction models indicates that accuracy is closely related to the formula and the data used for modeling and expression on a global scale. The latest models are globally expressed in the form of a grid, with a higher horizontal resolution and temporal resolution of data used for modeling to improve the overall accuracy. Researchers strive to develop models with higher accuracy and spatiotemporal resolution. TropGrid2 and GPT2 are the two most commonly used models. TropGrid2 adds diurnal variation to improve temporal resolution; however, ignorance of semi-annual variations introduces error. Although GPT2 considers semi-annual variations, it does not consider diurnal periodicity and therefore does reflect diurnal variations.

This study proposes a new tropospheric correction model ITG, based on ECMWF ERA-Interim output. Results show that ITG has better model formula and can provide temperature, pressure, Tm and ZWD. The paper is organized as follows: the construction of the proposed ITG model, followed by the accuracy evaluation, some conclusions are provided in the last section.

## Construction of ITG

### Expression of ITG

ITG is an improved model of GPT2 and TropGrid2 with a better model formula. It includes annual, semi-annual and diurnal variations as equation (1), where 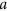 is the estimated tropospheric parameters (temperature, pressure, ZWD and so on), 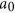 is the mean values; 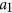, 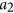 and 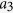 are the amplitudes of annual, semi-annual and diurnal periodicity, respectively; 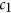, 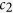 and 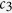 are the initial phase of annual, semi-annual and diurnal periodicity, respectively; *doy* is the day of the year, *hod* is the hour of the day. The amplitude and initial phase of diurnal variation are estimated as periodic functions with annual and semi-annual periodicity. Take the amplitude and initial phase of diurnal variation 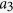 as an example to expand it as equation (2). Fifteen model coefficients of ITG improve temporal resolutions over GPT2 and TropGrid2. ITG is globally expressed in the form of a grid, and the resolution can be adjusted as needed. After obtaining four gridded coefficients around the station, the values at the station can be calculated with bilinear interpolation:
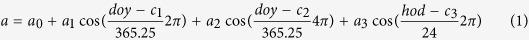

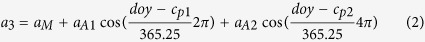
ITG uses the temperature lapse rate, equation (3) and equation (4) to convert temperature, pressure, and ZWD at the surface to the desired height, respectively.
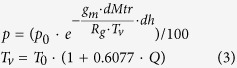

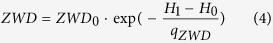
In equation (3), *p* is the pressure (hPa) at height *dh*, 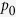 is the surface pressure, 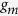 is the average gravity coefficient, 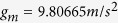,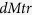 is the molar mass of dry air, 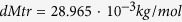,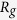 is the universal gas constant, 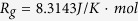 , 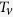 is the virtual temperature(Kelvin),
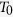
is the surface temperature, and *Q* is the specific humidity. In equation 4, *H*_*0*_ is the geopotential height of the grid-point *ZWD*_*0*_, *H*_*1*_ is the target height, the estimated *ZWD* is the ZWD at *H*_*1*_, and *q*_*ZWD*_ is the ZWD scale height, which is usually around 2 km.

### Data source for constructing ITG

Both ECMWF and NCEP provide products of NWM. GPT2 chooses ECMWF while TropGrid2 chooses NCEP. Several studies have compared the accuracy of ECMWF and NCEP. Yu *et al.*^27^ used meteorological measurements in Antarctic to test the accuracy of ECMWF ERA-40 and NECP-NCAR. Results showed that products of ECMWF are more accurate. Chen *et al.*^28^ compared the measured ZTD at 28 GPS tracking stations in China with calculated ZTD by using NWM from ECMWF and NCEP. Results indicate that the bias and standard deviation of ECMWF were more optimistic than NCEP, and closer to the measured ZTD. Observations on the global network were employed to test the accuracy of NCEP, ECMWF and GSFC (Goddard Space Flight Center) by Decker *et al.*^29^. Integrated results indicate that ECMWF ERA-Interim has the highest accuracy. Chen *et al.*^30^ used the measured ZTD at 49 GPS stations in Asia to assess ECMWF ZTD and NCEP ZTD. Compared with GPS ZTD, Bias and RMS for ECMWF ZTD were −1.0 cm and 2.7 cm, NCEP ZTD were 2.4 cm and 6.8 cm, and ECMWF ZTD was superior to NCEP ZTD.

ECMWF and NCEP have advantages and disadvantages. We used ECMWF ERA-Interim for modeling in this work. Its temporal resolution is 6 hours (at UTC 00:00, 06:00, 12:00, and 18:00), and all parameters in GPT2 are modeled with 37 layers of product for ECWMF ERA-Interim. We used surface products of ECMWF ERA-Interim to model surface parameters. We modeled the temperature lapse rate with 37 layers of product.

### Calculation of ITG coefficients

**Figure 1:**
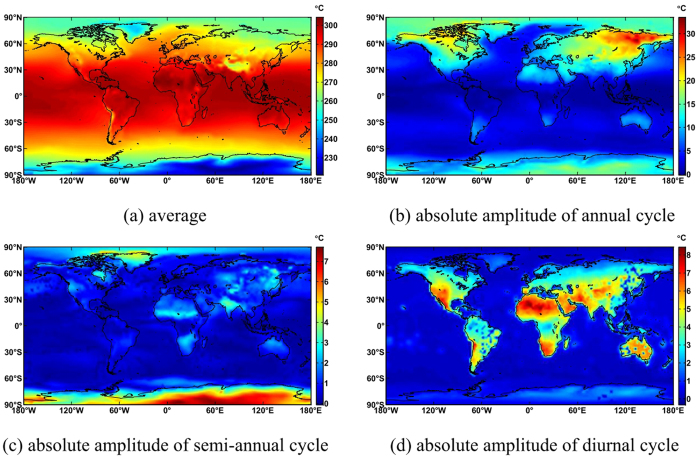
Global distribution of ITG temperature coefficients. The abscissa represents longitude, and the ordinate represents latitude. The globe is divided into a grid of 2.5 × 2.5, the value of each grid point are represented by the point color, the value each color represent are shown in the right of the figure. This figure is drawn using MATLAB software.(**a**) average (**b**) absolute amplitude of annual cycle (**c**) absolute amplitude of semi-annual cycle (**d**) absolute amplitude of diurnal cycle.

**Figure 2:**
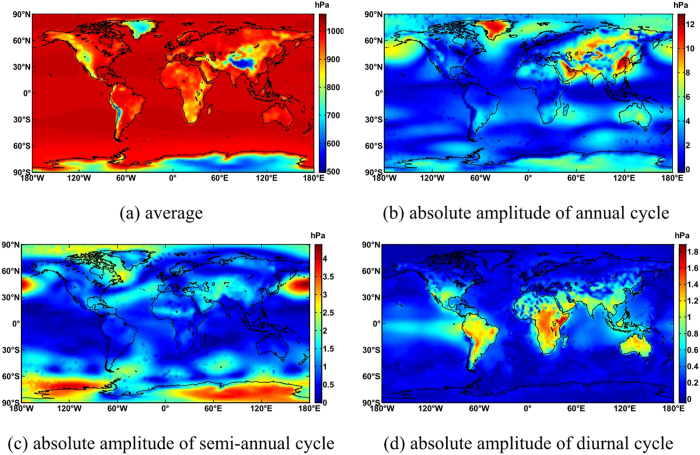
Global distribution of ITG pressure coefficients. This map was generated as the same way with Fig. 1(**a**) average (**b**) absolute amplitude of annual cycle(**c**) absolute amplitude of semi-annual cycle (**d**) absolute amplitude of diurnal cycle.

**Figure 3:**
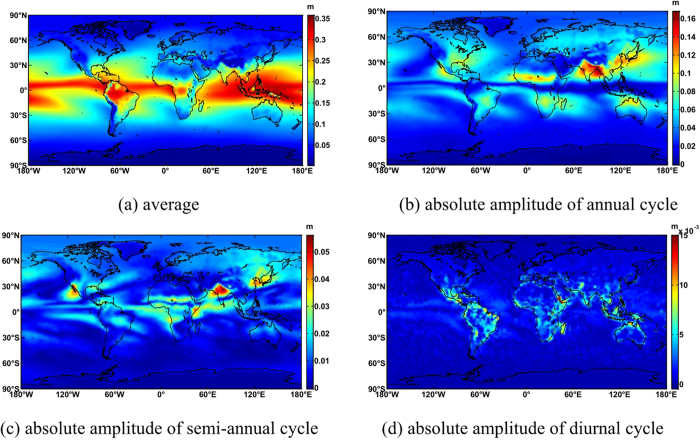
Global distribution of ITG ZWD coefficients. This map was generated as the same way with Fig. 1(**a**) Bias of ITG (**b**) RMS of ITG (**c**) Bias of GPT2 (**d**) RMS of ITG – GPT2 (**e**) Bias of GPT2 1 ×1  (**f**) RMS of ITG – GPT2 w 1 ×1 .

Parameters of ITG include temperature, pressure, Tm, ZWD and temperature lapse rate. They are modeled with the output of ECMWF ERA-Interim in 2001-2010 (spatial horizontal resolutions are 2.5° × 2.5°, temporal resolution is 6 hours) as the method introduced in section 3.2. From this, we can obtain the global gridded coefficients of temperature, pressure, Tm, ZWD and temperature lapse rate at the surface. Taking temperature, pressure and ZWD, for example, the global distribution of several coefficients are shown in figs. 1, 2, 3.

Figure 1 shows that the average coefficients of ITG for temperature decrease as latitude and altitude increase. They reach the minimum in the Antarctic and the maximum near the equator. Amplitudes of annual periodicity increase as latitude increases, and are larger on land than in sea areas, with the minimum in the equator and the maximum in Siberia. This is because differences in annual temperature are significant in Siberia and slight at the equator. The polar day and polar night in the polar region result in maximal semi-annual periodicity. The semi-annual periodicity in India is also large due to monsoons. Amplitudes of diurnal periodicity are closely associated with temperature differences between day and night. They are larger on land than in sea areas, especially in deserts, and reach a maximum in the Sahara. Fig. 2 shows that the average pressure decreases significantly as altitude increases, and it is much smaller in the polar regions, the Andes and Tibetan Plateau than in other areas. The amplitudes of annual periodicity are significant in parts of Asia and Greenland. The amplitudes of semi-annual periodicity are large in the North Pacific and Antarctic, while the amplitudes of diurnal periodicity occur at mid and low latitudes.

Figure 3 shows that the average ZWD increases as latitude and altitude decrease, and the maximum ZWD is near the equator. Amplitudes of annual periodicity are larger in the northern hemisphere than in the southern hemisphere, and reach a maximum in India and the Bay of Bengal. This is because the typical tropical monsoon results in an obvious difference in wet and dry seasons. Influenced by monsoons, the amplitudes of annual periodicity are considerable in eastern China, Korean Peninsula and southern of Japan. Slight annual variations at the equator contribute to a small amplitude of annual periodicity. Amplitudes of semi-annual periodicity are large in northern India, northern Mexico and eastern China, with a maximum in northern India. Amplitudes of diurnal periodicity occur mainly in coastal lands globally.

## Accuracy assessment

To further analyze the effectiveness and applicability of ITG, external references were used to compare the accuracy of the proposed ITG and the existing models in this section. The external references include the real meteorological records from NOAA, the GGOS Tm grids and the IGS ZTD products. The comparison is carried out in terms of temperature, Tm and ZTD. Some existing models include GPT2 series, TropGird2, EGNOS, UNB series and so on. However, the TropGrid2 products are unavailable to the public. On the other hand, the EGNOS and UNB series for ZTD have been shown to be inferior to GPT2. Therefore, in this study we use GPT2 series for the comparison. More specifically, two GPT2w versions with the horizontal resolutions of 1° × 1° and 5° × 5° are adopted.

First, we used observations from 698 globally distributed stations provided by NOAA as a reference to validate ITG, GPT2 and GPT2w for temperature. Average results are shown in Table 1. ITG showed the highest accuracy, with a bias of −0.27 °C and an RMS of 3.87 °C. Compared with the widely used GPT2, RMS improved to 0.93 °C, with a smaller bias. GPT2w of 5° × 5° was equivalent in accuracy to GPT2. Compared with GPT2w of 5° × 5°, GPT2w of 1° × 1° only improves RMS of 0.05 °C. Although GPT2w 1° × 1° uses higher spatial resolution data for modeling, the accuracy improvement for temperature was negligible.

**Figure 4:**
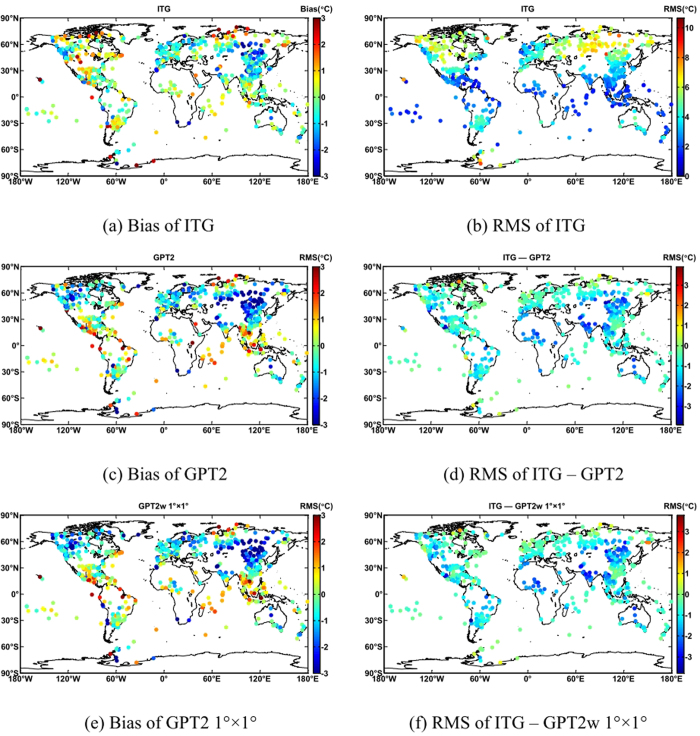
Global distribution of temperature RMS difference for each model. The abscissa represents longitude, and the ordinate represents latitude. 698 globally distributed stations provided by NOAA are represented by colorful dots, the color of each dot represent the Bias or RMS value at this station. This figure is drawn using MATLAB software.(**a**) Bias of ITG (**b**) RMS of ITG.

Figure 4(a) shows that the temperature in the northern of Central Asia and most of East Asia was lower than ever all year due to the influence of Arctic oscillation in 2012. Biases at many ​​stations were below -3 °C, and most stations in Europe exhibited negative biases. While biases in Arctic and North America were generally positive, some were larger than 2 °C. Fig. 4(b) shows that RMS increases as latitude increases. The majority of stations at low latitudes have an RMS of less than 3 °C. Most of the mid-latitude stations have an RMS of 3 °C ~ 6 °C. Some high latitude stations had an RMS larger than 6 ° C. Figs. 4(c) indicate that the GPT2 series have a larger bias than ITG. This results from different schemes for modeling, and ITG was found to be optimal. Figs. 4(d) show that most stations had a negative RMS, indicating that ITG is superior to the GPT2 series. Results for ITG were striking: it was superior to GPT2 at 93.1% of the stations and superior to GPT2 w of 1° × 1°at 93.1% of the stations.

ZTD products in 2012 at 280 IGS tracking stations were utilized for testing, with a temporal resolution of one hour. GNSS ZTD was used by Boehm *et al.*^2^ to test GPT2w with a temporal resolution of 6 hours. With higher temporal resolution testing data and yearly statistical results are shown in Table 2. ITG had the smallest bias at only 0.04 cm, and a range from -2.66 cm to 3.31 cm. Biases for GPT2 widely ranged from -7.32 cm to 8.29 cm. GPT2w of 5° × 5° had an average bias of -0.11 cm, and a maximal absolute bias of 5.54 cm.

Compared with GPT2w of 5° × 5°, GPT2w of 1° × 1° had a smaller maximum absolute bias of 4.57 cm, but a larger bias than ITG. ITG had an average RMS of 3.73 cm, smaller than GPT2 and GPT2w of 5° × 5°, and its accuracy was similar to GPT2w of 1° × 1°. However, the maximal RMS of ITG is smaller than GPT2w of 1° × 1°. Comparison between GPT2w of 5° × 5° and GPT2w of 1° × 1° shows that higher horizontal resolution improves the ZTD RMS of only 0.05 cm. Comprehensively, the ZTD from ITG is much closer to the IGS ZTD. Although GPT2w of 1° × 1° had a higher horizontal resolution, the formula and modeling method of ITG was shown to be superior. Hence, ITG performs better than GPT2w of 1° × 1°.

**Figure 5:**
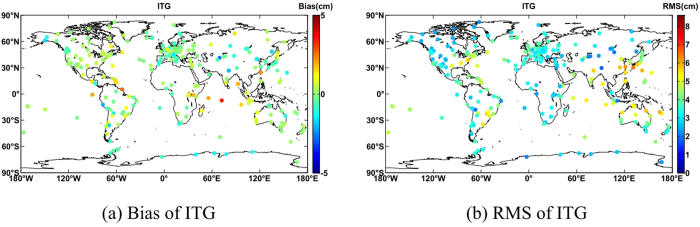
Global distribution of ZTD Bias/RMS difference for ITG. This map is generated as the same way with Fig. 4.

Figure 5(a) shows that biases are more likely to be negative at stations inland and in Antarctica, while they are positive at the sea or nearby. Fig. 5(b) indicates that RMS at high latitudes is generally smaller than at lower latitudes. Stations in Japan, eastern China, northern Australia, eastern America and southeast South America exhibit an RMS of larger than 4 cm, while most stations in Europe have an RMS of less than 4 cm. In addition, 64% of the stations overall had an RMS of less than 4 cm.

High accuracy Tm grids are provided by GGOS from ECMWF reanalysis data, and radiosonde data is utilized to verify the high accuracy of GGOS Tm by Yao *et al.*^16^. Therefore, the GGOS Tm in 2012 were employed to test ITG and GPT2 w, with annual statistical results are shown in Table 3. Biases for ITG range from -10.57 K to 2.10 K. The biases of GPT2w of 1° × 1° range from -11.59 K to 3.03 K, while ITG features the smallest bias. GPT2w of 5° × 5° has a maximum absolute bias of 24.2 K. Large biases indicate abnormality in some areas, since Tm is closely related with height. GPT2w of 5° × 5° shows low accuracy at the interpolated height, and the absence of elevation correction results in large deviations. ITG had an RMS of only 3.07 K, and was superior to the two GPT2 w versions. Comprehensively, Tm calculated by ITG showed the highest precision. It can guarantee the accuracy needed for converting ZWD to PWV.

## Conclusions

The tropospheric correction model has received considerable study, providing tropospheric delay corrections for GNSS. This can also be applied to weather forecast, climate change and other topics. The relevance and difference between tropospheric parameter measurements and estimated ones in convective weather can provide more references for weather forecasting. Variations of model coefficients in different periods contribute to further analysis of climate trends.

In this study, we developed a new model ITG which showed superior performance over the existing GPT2 series and TropGrid2. Numerical results indicate the proposed ITG outperforms the other models in terms of temperature, ZTD and Tm. More specifically, high-precision empirical temperature can be used for determining annual thermal deformations of radio telescopes (or for buildings with GNSS antennas on top of them), as reference temperatures for these thermal deformations^23^ or be used in the study of climate change. Regarding the ZTD, ITG can provide tropospheric delay corrections with a bias of 0.04 cm and an RMS of 3.73 cm. As for the mean weighted temperature, Tm provided by ITG guarantees the conversion accuracy from ZWD to PWV and the application of GNSS vapor monitoring. Multi-source data fusion for modeling is relevant to our research, and is likely to improve accuracy further.

## Additional Information

**How to cite this article**: Yao, Y. *et al.* ITG: A New Global GNSS Tropospheric Correction Model. *Sci. Rep.* **5**, 10273; doi: 10.1038/srep10273 (2015).

## Figures and Tables

**Table 1:** Statistical results of temperature bias/RMS for each model (NOAA).

**Model**	**Bias (°C)**			**RMS (°C)**		
	**Mean**	**Max**	**Min**	**Mean**	**Max**	**Min**
ITG	−0.27	6.84	−9.14	3.87	10.66	0.69
GPT2	−0.46	8.17	−7.09	4.80	12.38	0.57
GPT2w 1°×1°	−0.45	5.43	−6.72	4.75	11.85	0.49
GPT2w 5°×5°	−0.46	8.17	−7.09	4.80	12.38	0.57

**Table 2:** Statistical results of ZTD Bias/RMS for each model (280 IGS stations)

**Model**	**ZTD Bias (cm)**			**ZTD RMS (cm)**		
	**Mean**	**Max**	**Min**	**Mean**	**Max**	**Min**
ITG	0.04	3.31	−2.66	3.73	6.46	1.86
GPT2	0.16	8.29	−7.32	4.33	8.55	1.80
GPT2w 1°×1°	−0.14	2.48	−4.57	3.73	6.52	1.92
GPT2w 5°×5°	−0.11	3.55	−5.54	3.78	6.88	1.95

**Table 3:** Statistical results of Tm Bias/RMS for each model (GGOS)

**Model**	**Bias (K)**			**RMS (K)**		
	**Mean**	**Max**	**Min**	**Mean**	**Max**	**Min**
ITG	−0.11	2.10	−10.57	3.07	10.98	0.98
GPT2w 1°×1°	−0.09	3.03	−11.59	3.24	12.03	0.96
GPT2w 5°×5°	−0.08	12.45	−24.20	3.38	24.45	0.98
